# Identification of a promising PI3K inhibitor for the treatment of multiple myeloma through the structural optimization

**DOI:** 10.1186/1756-8722-7-9

**Published:** 2014-01-15

**Authors:** Kunkun Han, Xin Xu, Guodong Chen, Yuanying Zeng, Jingyu Zhu, Xiaolin Du, Zubin Zhang, Biyin Cao, Zhaopeng Liu, Xinliang Mao

**Affiliations:** 1 Cyrus Tang Hematology Center, Soochow University, Suzhou, Jiangsu Province 215123, China; 2 Department of Organic Chemistry, Key Laboratory of Chemical Biology (Ministry of Education), School of Pharmaceutical Sciences, Shandong University, Jinan 250012, Shandong Province, China; 3 Department of Pharmacology, School of Pharmacy, Soochow University, Suzhou, Jiangsu Province 215123, China

**Keywords:** Phosphatidylinositol 3-kinase (PI3K)/AKT signaling pathway, BENC-511, S14161, Multiple myeloma, Drug discovery

## Abstract

**Background:**

We previously reported a PI3K inhibitor S14161 which displays a promising preclinical activity against multiple myeloma (MM) and leukemia, but the chiral structure and poor solubility prevent its further application.

**Methods:**

Six S14161 analogs were designed based on the structure–activity relationship; activity of the compounds in terms of cell death and inhibition of PI3K were analyzed by flow cytometry and Western blotting, respectively; anti-myeloma activity *in vivo* was performed on two independent xenograft models.

**Results:**

Among the six analogs, BENC-511 was one of the most potent compounds which significantly inhibited PI3K activity and induced MM cell apoptosis. BENC-511 was able to inactivate PI3K and its downstream signals AKT, mTOR, p70S6K, and 4E-BP1 at 1 μM but had no effects on their total protein expression. Consistent with its effects on PI3K activity, BENC-511 induced MM cell apoptosis which was evidenced by the cleavage of Caspase-3 and PARP. Notably, addition of insulin-like growth factor 1 and interleukin-6, two important triggers for PI3K activation in MM cells, partly blocked BENC-511-induced MM cell death, which further demonstrated that PI3K signaling pathway was critical for the anti-myeloma activity of BENC-511. Moreover, BENC-511 also showed potent oral activity against myeloma *in vivo*. Oral administration of BENC-511 decreased tumor growth up to 80% within 3 weeks in two independent MM xenograft models at a dose of 50 mg/kg body weight, but presented minimal toxicity. Suppression of BENC-511 on MM tumor growth was associated with decreased PI3K/AKT activity and increased cell apoptosis.

**Conclusions:**

Because of its potent anti-MM activity, low toxicity (LD_50_ oral >1.5 g/kg), and easy synthesis, BENC-511 could be developed as a promising agent for the treatment of MM via suppressing the PI3K/AKT signaling pathway.

## Introduction

The phosphatidylinositol 3-kinases (PI3Ks) are a family of intracellular lipid enzymes that phosphorylate the 3′-OH group at the inositol ring of phosphatidylinositol and convert PI(4,5)P2 to PI(3,4,5)P3 [1,2]. The latter then acts as a secondary messenger that mediates the AKT activation and a series of downstream signals that are responsible for various cell activities, such as tissue factor expression and coagulation [3], cell proliferation and survival [4]. Dysregulation of the PI3K/AKT signaling pathway is frequently seen in many cancer types [5-7], including hematological malignancies, such as leukemia [8], lymphoma [9], and multiple myeloma (MM) [10,11]. Overactivation of PI3K/AKT confers chemoresistance and poor outcomes, while knockdown of PI3K or AKT leads to cancer cell death [5,12,13]. Therefore, the PI3K/AKT pathway is regarded as an ideal target for cancer therapy [5-7]. Actually, more and more PI3K/AKT inhibitors have been identified, of which some have been successfully moved into clinical evaluation [14,15].

MM is a malignancy of plasma cells and it accounts for more than 10% of all hematological cancers and 2% of annual cancer-related death [16]. It is believed that the PI3K/AKT pathway is particularly pertinent for MM growth and therapy. There are four members in the Class I PI3K family, namely, PI3Kα, β, δ and γ, all of which are overactivated in MM cell lines and primary myeloma patient cells [17]. Moreover, phosphatase and tensin homolog (PTEN), the critical negative modulator of PI3K signaling, is frequently deleted or inactivated by mutation in MM cells [18]. Activation of PI3Ks in MM is associated with growth factors such as insulin-like growth factor 1 (IGF-1) and cytokines such as interleukin-6 (IL-6), both of which are highly expressed in MM cells [19]. In an analysis of AKT activity in MM cells, the expression of phosphorylated-AKT (S473) was found in 16 of 18 patients, which indicates constitutively phosphorylated-AKT in primary MM cells [20]. In addition, this key signaling is also an indicator of unfavorable outcomes of myeloma patients [20]. Moreover, inhibition of PI3K/AKT leads to MM apoptosis. Therefore, PI3K/AKT is an ideal target for anti-myeloma drug discovery. Many inhibitors of the PI3K/AKT signaling pathway, such as CAL-101, NVP-BKM120, and Perifosine have been developed for MM therapy and are now evaluated in clinical trials [21-23]. However, there are no approved PI3K inhibitors available for MM therapy due to collective efficacy and/or safety issues, novel PI3K inhibitors are in demand.

We recently identified S14161 as an inhibitor of pan-Class I PI3K isoforms by a high throughput screening strategy [11]. S14161 induces apoptosis in myeloma and leukemia cell lines as well as primary patient samples. Notably, S14161 is also effective in leukemia xenograft models [11]. Given its potent antileukemia and antimyeloma activity and minimal toxicity, S14161 can serve as a new lead for cancer drug development. To improve its physical and chemical properties, such as solubility and chirality, we designed a series of analogs of S14161 based on the structure-activity relationship, and identified BENC-511 as a more potent inhibitor of PI3K signaling for the treatment of MM.

## Results

### BENC-511 displays potent inhibitory effects on AKT activation

**Figure 1:**
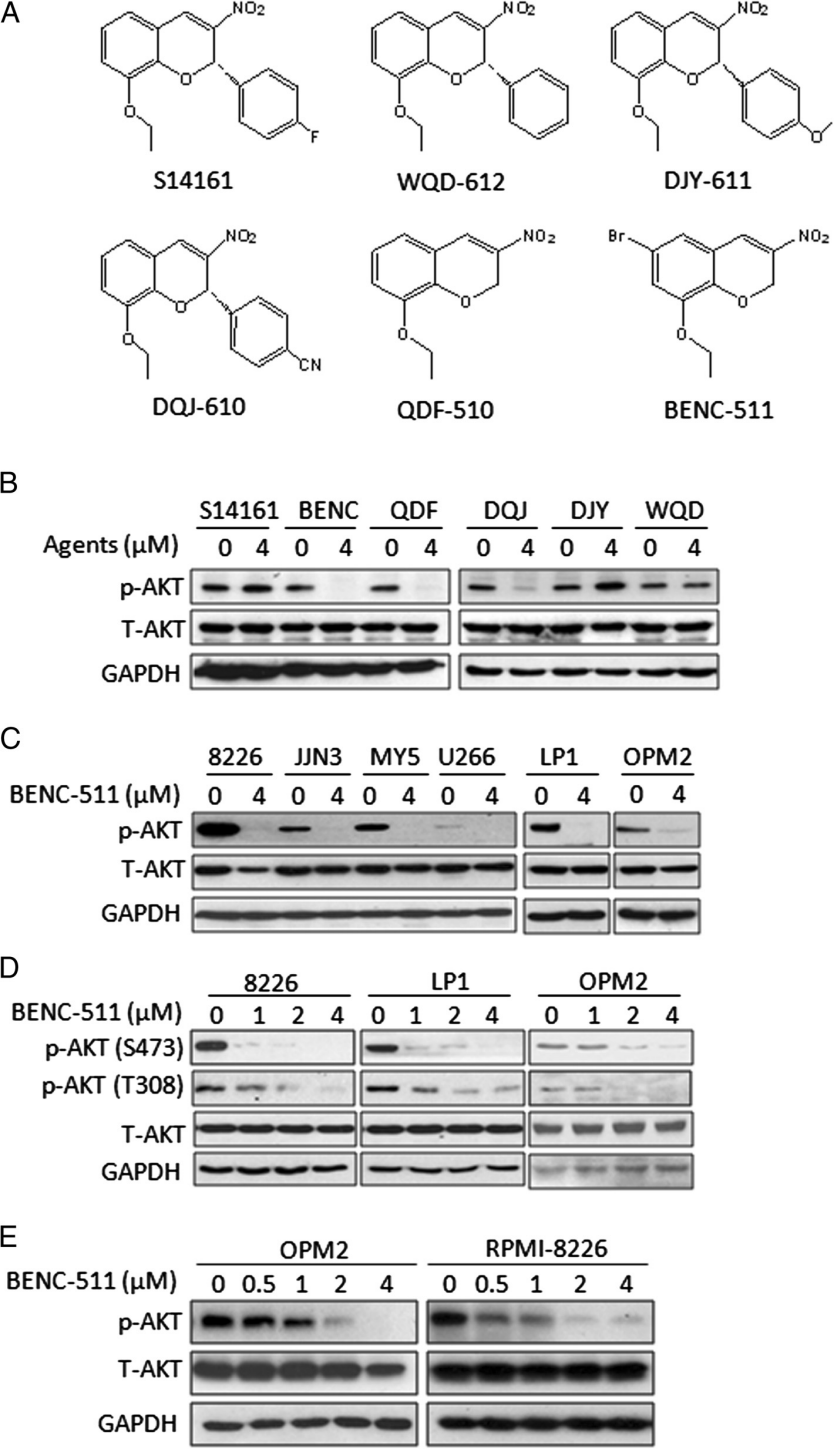
**BENC-511 displays potent inhibitory effects on AKT activation. (A)** The structures of the analogs of S14161, including DQJ-610, DJY-611, WQD-612, QDF-510, BENC-511. **(B)** OPM2 cells were treated with 4 μM of S14161, BENC (BENC-511), QDF (QDF-510), DQJ (DQJ-610), DJY (DJY-611), or WQD (WQD-612) for 24 hours. After incubation, cells were harvested and total proteins were isolated. Expression of p-AKT (S473) and total AKT were measured by immunoblotting. **(C)** RPMI-8226 (8226), JJN3, OCI-MY5 (MY5), U266, LP1, OPM2 cells were treated with 4 μM of BENC-511 or DMSO for 24 hours followed by the analysis of the expression of p-AKT (S473) and total AKT. **(D)** RPMI-8226, LP1 and OPM2 cells were treated with increasing concentration of BENC-511 for 24 hours. Expression of p-AKT (S473 and T308), and total AKT were measured by immunoblotting. **(E)** RPMI-8226 and OPM2 cells were treated with increasing concentration of BENC-511 for 12 hours followed by the analysis of AKT activation. GAPDH was used as a loading control. T-AKT: total AKT.

We previously reported S14161 as a novel PI3K inhibitor [11], to improve its physical and chemical properties, we designed two classes of analogs of S14161. Class I members WQD-612, DJY-611, DQJ-610 contained the 2-phenyl ring, but the flouro substituent was replaced with a hydrogen atom or an electron-withdrawing cyano group or electron-donating methoxy substituent at the para position (Figure 1A). Class II members QDF-510 and BENC-511 were designed with a simplified structure by removing the 4-fluorophenyl group at the 2-position of the chromene core (Figure 1A). In the analysis of the effects of these analogs on PI3K activity, we treated the PTEN-negative MM cell line OPM2 with 4 μM of each compound for 24 hours. Western blotting analyses revealed that all class I compounds except DJY-611 showed no significant inhibitory effects on AKT phosphorylation, while the class II compounds effectively suppressed AKT activation (Figure 1B). We next evaluated the inhibitory effects of these compounds on OPM2 cell growth. Over a 72-hour treatment, BENC-511 was found to be the most potent one in inhibiting OPM2 cell proliferation by a measurement of viable cells using MTT assay (Additional file 1: Figure S1). BENC-511 was then applied for evaluation of AKT phosphorylation levels in a panel of MM cell lines, including RPMI-8226, JJN3, OCI-MY5, U266, LP1, and OPM2. As shown in Figure 1C, BENC-511 inhibited AKT activation in all cell lines examined.

To further characterize the effect of BENC-511 on AKT activation, BENC-511 was added to RPMI-8226, LP1, and OPM2 cells from 1 to 4 μM for 24 hours. Western blotting analysis revealed that AKT phosphorylation was inhibited at both T308 and S473 sites in a concentration- and time- dependent manner (Figure 1D and E). Because the full activation of AKT depends on both T308 and S473 sites, these results suggested that BENC-511 fully suppressed AKT activation at high concentrations.

### BENC-511 suppresses AKT activation triggered by IGF-1 and IL-6

**Figure 2:**
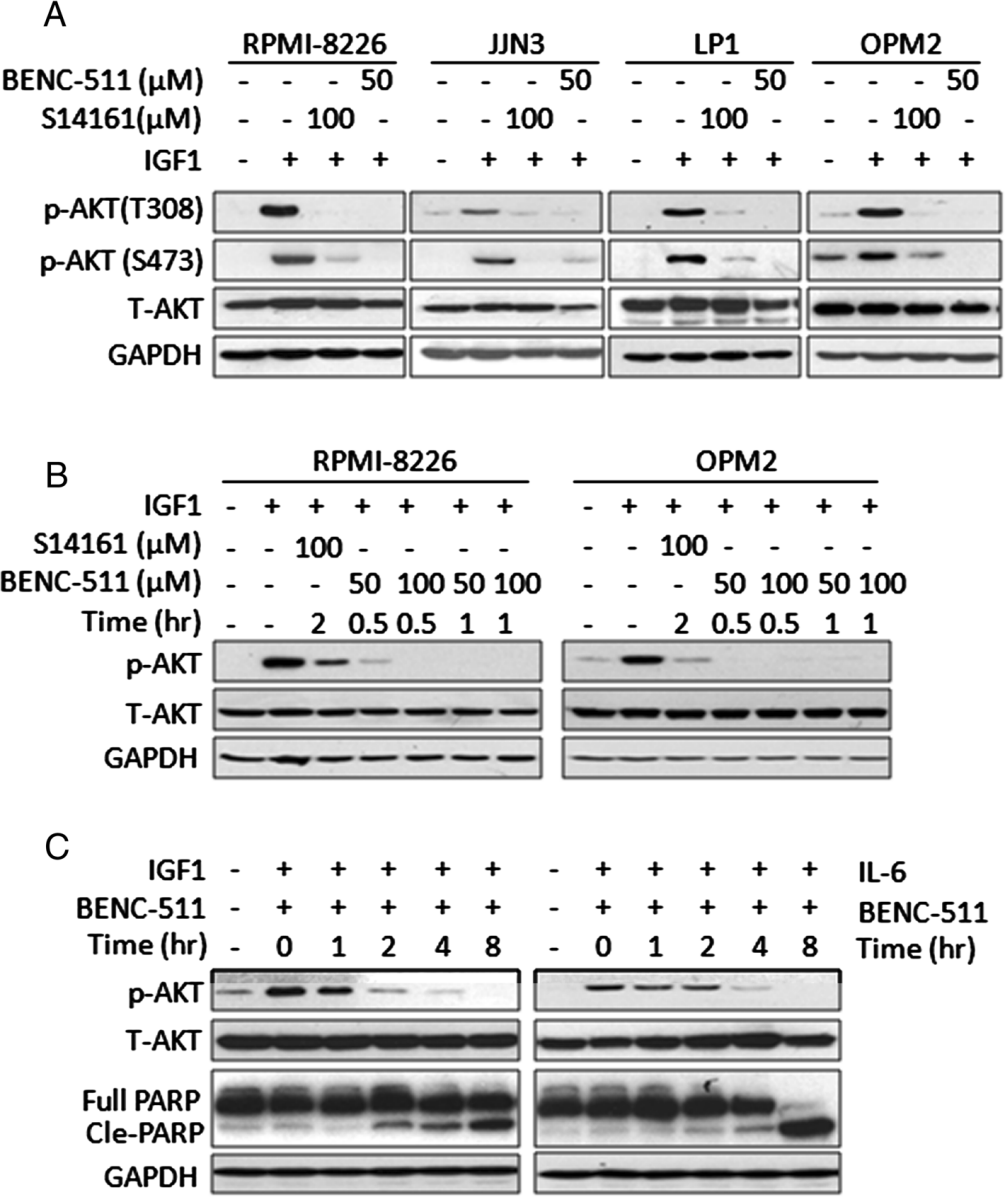
**BENC-511 suppresses AKT activation triggered by IGF-1. (A)** Myeloma (RPMI-8226, JJN3, LP1 and OPM2) cells were starved overnight and then treated with 100 μM of S14161, 50 μM of BENC-511, or DMSO for 2 hours, followed by 100 ng/mL of IGF-1 for 15 minutes. After incubation, cells were harvested and total proteins were isolated. Expression of AKT, p-AKT (T308), p-AKT (S473), and GAPDH was measured by immunoblotting. **(B)** RPMI-8226 and OPM2 cells were treated with increasing concentration of BENC-511 for 0.5 or 1 hour, S14161 for 2 hours, followed by IGF-1 stimulation. Cells were then harvested and total proteins isolated. Expression of AKT, p-AKT (S473), and GAPDH was measured by immunoblotting. **(C)** OPM2 cells were starved overnight and then treated with BENC-511 (8 μM), or DMSO for the indicated time followed by 100 ng/mL IGF-1 or 50 ng/mL IL-6 for 15 minutes. After incubation, cells were harvested and total proteins isolated. Expressions of total AKT, p-AKT (S473), PARP, and GAPDH were measured by immunoblotting. T-AKT: total AKT.

One of the biological signals that activate PI3K/AKT pathway is from receptor tyrosine kinases [15]. In MM cells, the most important signal stimulators are IGF-1 and IL-6 which regulate MM cell growth, proliferation and angiogenesis [24]. To evaluate the effects of BENC-511 on AKT activation, 4 MM cell lines RPMI-8226, OPM2, JJN3 and LP1 were starved overnight and then treated with S14161 (100 μM) or BENC-511 (50 μM) for 2 hours, followed by stimulation with 100 ng/mL of IGF-1. The phosphorylation level of AKT at T308 and S473 were measured. It showed that BENC-511 suppressed AKT activation at both sites in the presence of IGF-1 (Figure 2A) and BENC-511 seemed more potent than S14161. As shown in Figure 2A, BENC-511 almost completely suppressed AKT phosphorylation at T308 in all cell lines, but there was a certain level of phosphorylated AKT remained in cells treated with S14161. To verify this effect, RPMI-8226 and OPM2 cells were treated with BENC-511 at 50 or 100 μM for 0.5 to 1 hour, immunoblotting assays revealed that 50 μM of BENC-511 suppressed AKT phosphorylation in 0.5 hour (Figure 2B), while AKT activation was observed after 100 μM of S14161 treatment for 2 hours. Because both cytokine IL-6 and growth factor IGF-1 are stimulators of PI3K activation and key regulators of MM cell growth, we next evaluated the effects of BENC-511 on AKT in the presence of IL-6 and IGF-1. In this experiment, starved OPM2 cells were treated with BENC-511 (8 μM) for 0–8 hours, followed by IL-6 or IGF-1 stimulation. As shown in Figure 2C, BENC-511 suppressed AKT activation raised by both IGF-1 and IL-6 within 2 or 4 hours. Notably, suppression of AKT phosphorylation was accompanied by PARP cleavage (Figure 2C), suggesting AKT suppression by BENC-511 was associated with MM cell apoptosis.

### BENC-511 downregulates PI3K/AKT downstream signals

**Figure 3:**
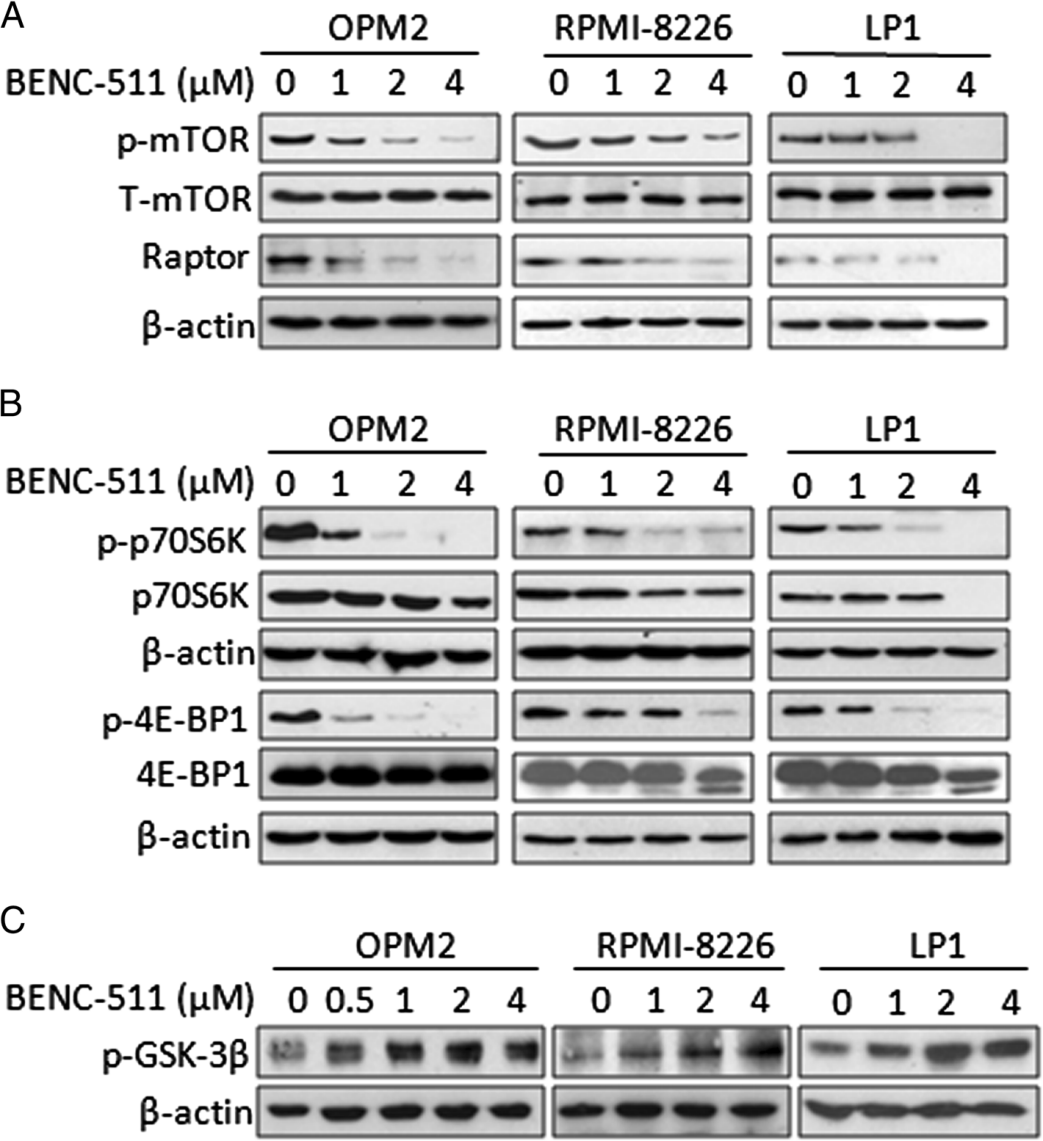
**BENC-511 downregulates PI3K/AKT downstream signals.** OPM2, RPMI-8226 and LP1 cells were treated with increasing concentrations of BENC-511 for 24 hours. Whole lysates were subjected to Western blot analysis. **(A)** p-mTOR (Ser2448), T-mTOR, Raptor; **(B)** p-p70S6K, p70S6K, p-4E-BP1, and 4E-BP1; **(C)** p-GSK-3β (Ser9). β-actin was used as an internal control.

As a central node of various cell signals, PI3K/AKT can regulate many key important signals, including the mammalian target of rapamycin (mTOR), protein 70S6 kinase (p70S6K), factor 4E binding protein 1 (4E-BP1), and glycogen synthase kinase-3, all of these proteins are key players in regulating protein synthesis and cell proliferation [25,26]. To evaluate the biological effects of BENC-511 on the PI3K/AKT signaling pathway, we measured the effects of BENC-511 on these protein phosphorylation levels. MM cells were treated with BENC-511 for 24 hours at indicated concentrations. Immunoblotting assays with specific antibodies demonstrated that BENC-511 inhibited expression levels of phosphorylated mTOR as well as its adaptor protein Raptor (Figure 3A), phosphorylated p70S6K and 4E-BP1 (Figure 3B). BENC-511 also induced GSK-3β activation as seen in its phosphorylation level (Figure 3C). These changes, consistent with previous reports on PI3K inhibitors [27], further demonstrated that BENC-511 was an inhibitor of PI3K.

### BENC-511 induces MM cell apoptosis

**Figure 4:**
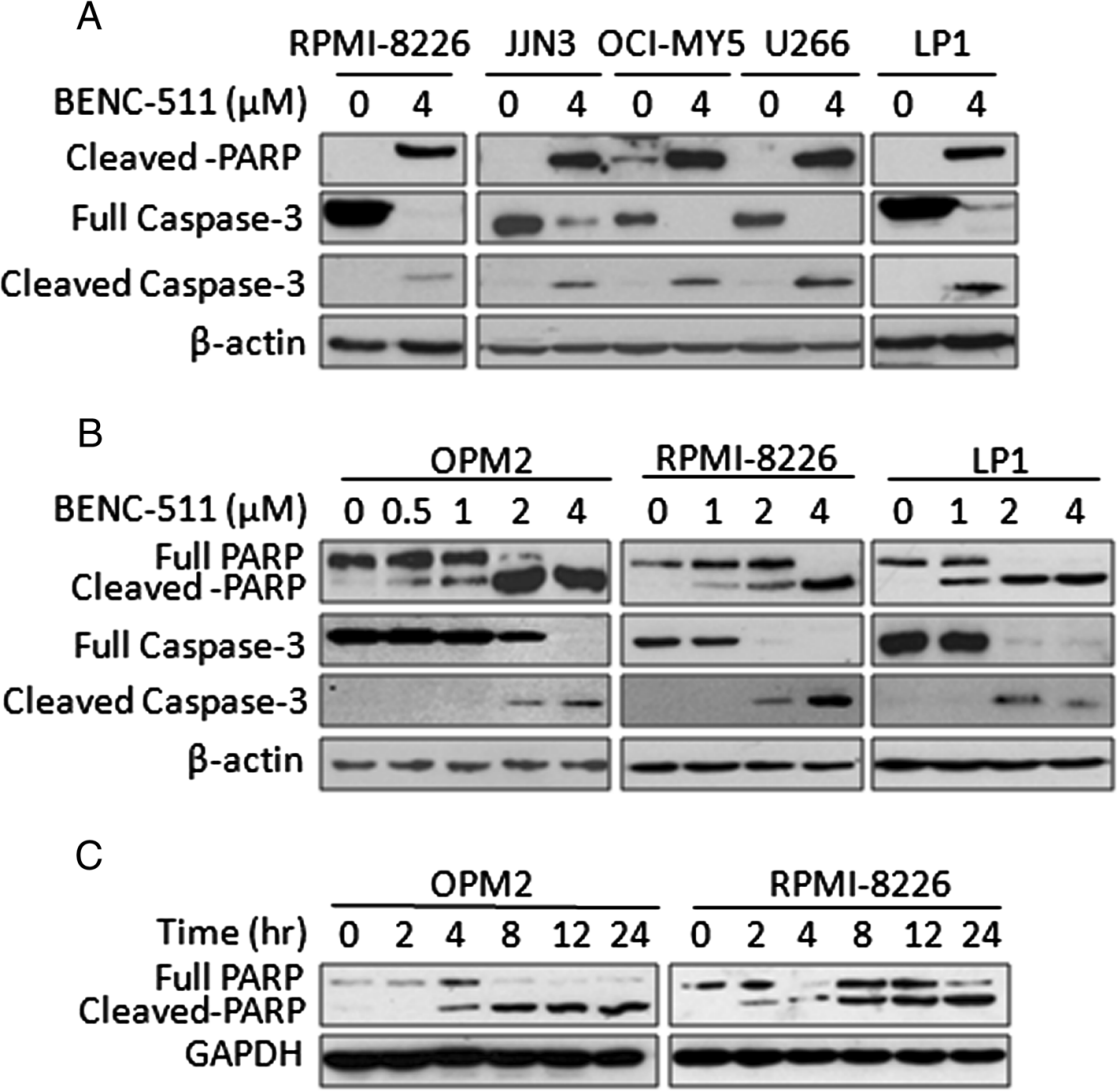
**BENC-511 activates apoptotic signaling in MM cells. (A)** Myeloma cells (RPMI-8226, JJN3, OCI-MY5, U266, LP1) were treated with 4 μM BENC-511 or DMSO for 24 hours followed by the analysis of the expression of PARP, Caspase-3, and GAPDH. **(B)** OPM2, RPMI-8226 and LP1 cells were treated with increasing concentrations of BENC-511 for 24 hours. **(C)** OPM2 and RPMI-8226 were incubated with 4 μM of BENC-511 for the indicated time. After incubation, cells were harvested and total proteins were isolated. Cell lysates were applied for PARP and GAPDH analyses.

Inhibition of PI3K/AKT results in apoptosis of cancer cells. S14161 has been demonstrated to induce MM cell death by targeting the PI3K signaling pathway in MM cells [11]. To investigate the effects of BENC-511 on MM cell apoptosis, we first evaluated the effects of BENC-511 on 5 MM cell lines. BENC-511 cleaved PARP and Caspase-3 (Figure 4A), and this effect was presented in a concentration- and time-dependent manner (Figure 4B). BENC-511 induced MM cell apoptosis at 0.5 μM within 24 hours (Figure 4B). A time-course study demonstrated that BENC-511 at 8 μM could cleave PARP within 2 and 4 hours in RPMI-8226 and OPM2 cells, respectively (Figure 4C).

**Figure 5:**
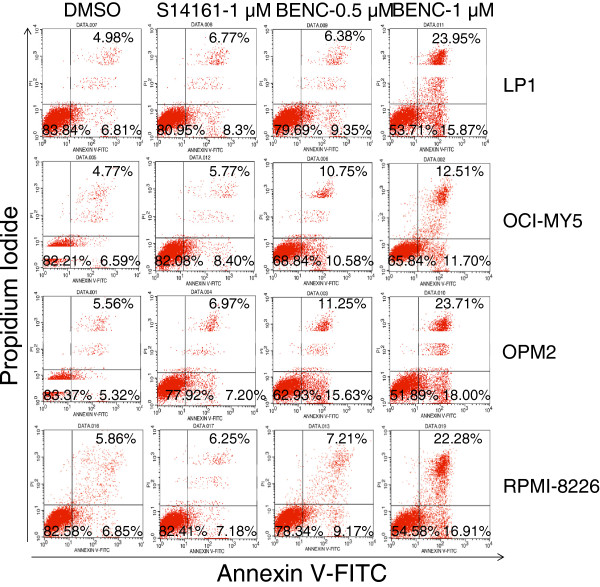
**BENC-511 induces MM cell apoptosis.** LP1, OCI-MY5, OPM2 and RPMI-8226 were treated with 1 μM of S14161, 0.5 or 1 μM of BENC-511 (BENC) and DMSO for 24 hours. Cells were then stained with Annexin V-FITC and PI and analyzed on a BD FACSCalibur™ flow cytometer.

To further demonstrate cell apoptosis, we measured cell apoptosis by Annexin-V and propidium iodide staining, where Annexin V specifically binds to phosphatidylserine on the surface of apoptotic cells while propidium iodide can penetrate into the dead cells and binds to the nuclei. Flow cytometric analyses revealed that BENC-511 at 1 μM induced more apoptotic cells than S14161 at 1 μM. For example, the apoptotic and dead fractions of LP1 cells was 15.87% and 23.95%, respectively, when treated with BENC-511, however, these fractions were only 8.3% and 6.77%, respectively, if treated with S14161 at the same concentration and incubation time (Figure 5). Therefore, BENC-511 was more potent than S14161 in cell apoptosis induction.

### BENC-511 induces MM cell apoptosis in the presence of IL-6 or IGF-1

**Figure 6:**
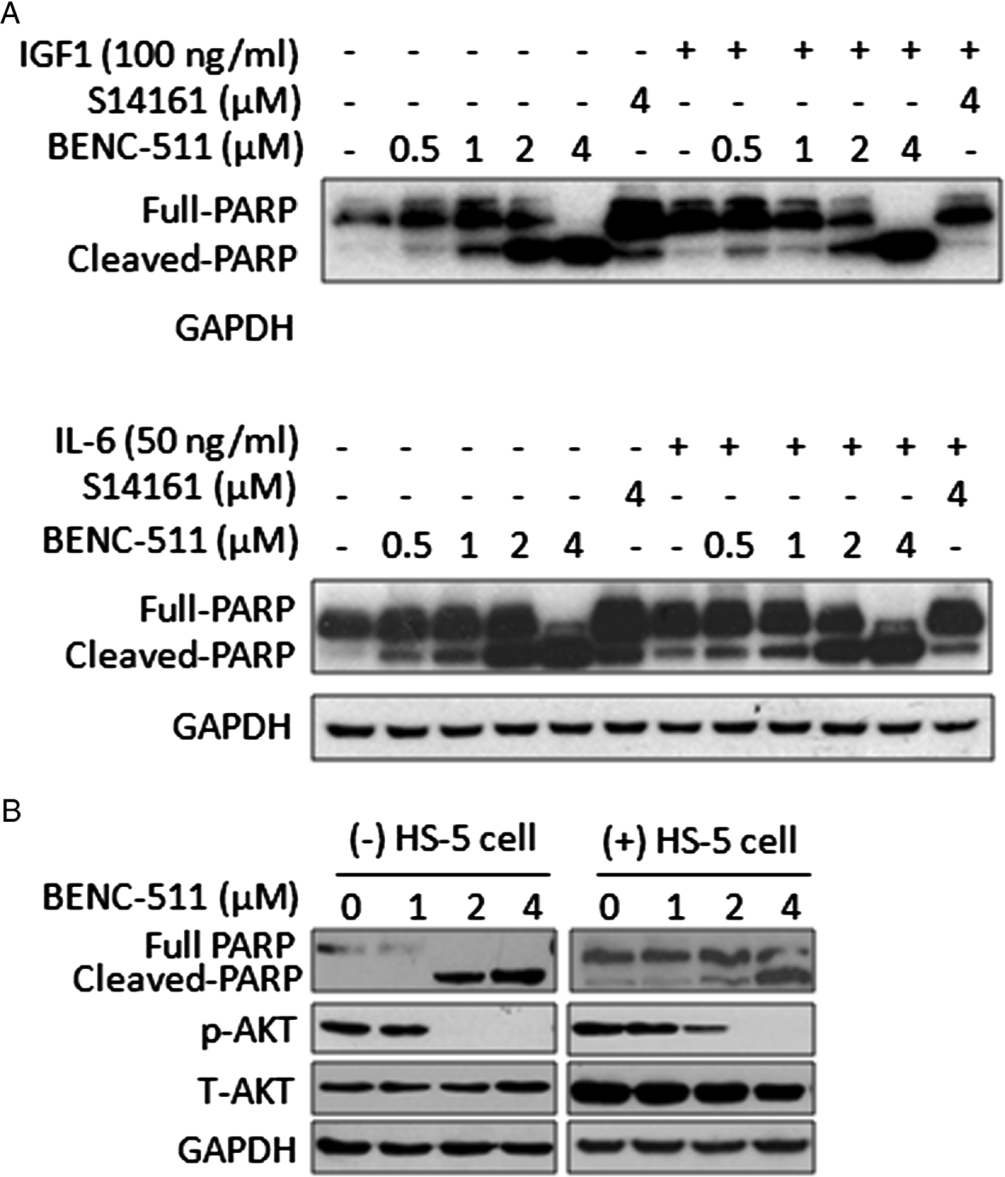
**BENC-511 induces MM cell apoptosis in the presence of IL-6 or IGF-1. (A)** OPM2 cells were co-treated with IL-6, IGF-1 and BENC-511 with the increasing concentration for 24 hours. After incubation, cells were harvested and cell lysates were measured for the expression of PARP and GAPDH by Western blotting. **(B)** MM cell lines OPM2 was co-cultured overnight with human bone marrow stromal cell HS-5, followed by treatment with BENC-511 overnight. Cells were then collected for PARP, p-AKT (S473) and total AKT (T-AKT) analysis.

As stated earlier, cytokines such as IL-6 and growth factors such as IGF-1 are key triggers of the PI3K/AKT signaling pathway, and critical regulators in MM cell proliferation. To find out whether BENC-511 inhibits PI3K activity is associated with its anti-myeloma activity, OPM2 cells were treated with BENC-511 from 0.5 to 4 μM or S14161 (4 μM) in the presence of IL-6 or IGF-1 for 24 hours. Apoptotic analyses with PARP suggested that BENC-511 markedly induced PARP cleavage at 1 μM, however, S14161 generated minimal effects at 4 μM. In the presence of IL-6 or IGF-1, cell death was partly blocked, especially at lower concentrations (Figure 6A). This finding demonstrated that BENC-511 induced MM cell apoptosis by targeting the PI3K signaling pathway.

IL-6 and IGF-1 can be secreted by the bone marrow stromal cells (BMSC) and regulate proliferation and survival of MM cells by regulating the PI3K/AKT signaling pathway via autocrine and/or paracrine manners [26,28]. To further demonstrate the effects of IGF-1 and IL-6 on MM cell growth, MM cell line OPM2 was cultured alone or in the presence of human stromal cell line HS-5. PARP cleavage assay revealed that stromal cells conferred resistance to BENC-511 because the presence of stromal cells attenuated the effects of BENC-511 on PARP cleavage and AKT activation in MM cells (Figure 6B). This further demonstrated that BENC-511 induced MM cell apoptosis in association with PI3K/AKT signaling.

### BENC-511 induces MM cell death *in vivo* and delays tumor growth in myeloma xenograft models

**Figure 7:**
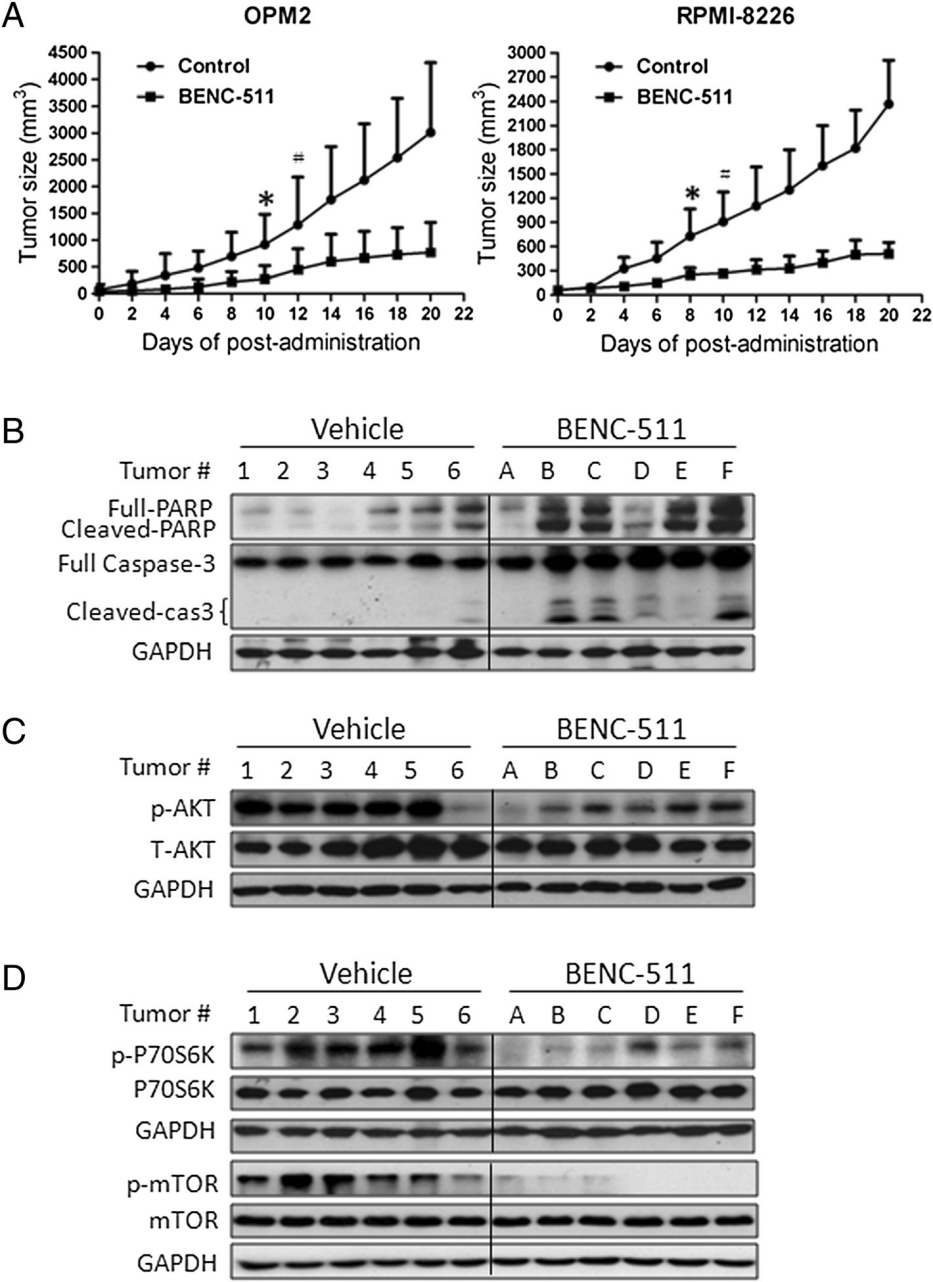
**BENC-511 induces MM cell death in vivo and delays tumor growth in myeloma xenograft models.** Human multiple myeloma cells (RPMI-8226 and OPM2) were injected subcutaneously into nude mice with a density of 30 million cells/site. When tumors were palpable, mice (n = 10/group) were orally given BENC-511 (50 mg/Kg body weight) in PBS containing 10% Tween 80 and 10% DMSO daily for continuous 20 days. Tumor volumes **(A)** were monitored every other day. *, p < 0.05; #, p < 0.01. Tumor samples from each group were subject to immunoblotting analysis for PARP, Caspase-3, phospho-AKT, T-AKT, p-mTOR, T-mTOR, p-p70S6K, and p70S6K with specific antibodies **(B, C,** and **D)**.

To further evaluate the therapeutic effects of BENC-511 in MM, two myeloma tumor models established with human MM cell lines OPM2 and RPMI-8226 in nude mice were treated with BENC-511 by oral administration. As shown in Figure 7A, BENC-511 at 50 mg/kg/day significantly decreased tumor growth within one week in both models. BENC-511 delayed MM tumor growth in a time-dependent manner. At the end of the experiment with 20-day treatment, the average tumor sizes were decreased to 25% and 21.2% compared with the control treated with vehicle in OPM2 and RPMI-8226 models, respectively (Figure 7A).

To check whether tumor decrease was associated with PI3K inhibition and apoptosis induced by BENC-511, tumor tissue extracts were subject to immunoblotting analyses. The results showed that both PARP and Caspase-3 were markedly cleaved in BENC-511 treated mice (Figure 7B). To our expectation, AKT phosphorylation was significantly suppressed in tumors from BENC-511-treated mice (Figure 7C). p70S6K and mTOR phosphorylation was also decreased in the same pattern as AKT (Figure 7D). However, BENC-511 had no changes in total protein levels in AKT, p70S6K, or mTOR (Figure 7). These data thus further demonstrated that BENC-511 was effective in the treatment of MM both *in vitro* and *in vivo*, which was highly associated with the suppression of the PI3K/AKT signaling pathway by BENC-511.

### BENC-511 displays minimal toxicity *in vivo*

Notably, there were no overt toxic changes in gross organs or body weight (Data not shown) after BENC-511 treatment up to 3 weeks (data not shown). To further evaluate the toxicity of BENC-511 *in vivo*, blood samples were taken from mice treated with BENC-511 or vehicles for 3 weeks and subject to hematology and biochemical analyses. The results showed that there were no significant changes in blood cell and platelet counts, and hemoglobin measurement (Table 1). Further evaluation focused on biochemical and enzymatic analyses that are associated with liver and kidney function, including aspartate aminotransferase (AST), alanine aminotransferase (ALT), blood urea nitrogen (BUN), and creatinine (Cr). Studies were performed based on 6 mice from each group and it showed that there were no marked changes in these biomarkers (Table 2). All these data thus suggest that BENC-511 is not toxic to liver and kidney. Taken together with the effects of body weight and blood analyses, BENC-511 was concluded as a non-toxic or minimal toxic chemical compound.

**Table 1:** Toxicology of BENC-511 in nude mice: hematology analysis

**Treatment**	**WBC (10**^**9**^**/L)**	**RBC (10**^**12**^**/L)**	**HGB (g/L)**	**PLT (10**^**9**^**/L)**
Control	1.81 ± 0.65	7.54 ± 1.37	110.00 ± 18.80	511.92 ± 134.68
BENC-511	1.70 ± 0.50	8.18 ± 1.97	123.80 ± 29.78	530.50 ± 112.82
*P*	0.89	0.68	0.47	0.58

Data represent mean ± SD of 6 nude in each group. Female nude mice (n = 6 mice per group) were treated with BENC-511 at 50 mg/kg body weight or vehicle for 20 days. Blood samples were collected in tubes containing EDTA as anticoagulant and subject to hematological analysis. WBC: White blood cell; RBC: Red blood cell; PLT: Platelets; HGB: haemoglobin.

**Table 2:** Toxicology of BENC-511 in nude mice: biochemical analysis

**Treatment**	**ALT, U/L**	**AST, U/L**	**BUN, mmol/L**	**Cr, μmol/L**
Control	62 (29.3 to 94.7)	237.7 (214.1 to 261.2)	10.3 (9.0 to 11.5)	10.7 (7.9 to 13.5)
BENC-511	48.2 (35.3 to 61.1)	189 (170.8 to 207.2)	11.3 (8.9 to 13.7)	12.4 (11.2 to 13.6)
*P*	0.6	0.3	0.3	0.4

Data represent mean ± SD of 6 nude in each group after treatment with BENC-511 at 50 mg/kg body weight or vehicle for 20 days. At the completion of the experiment, mice were sacrificed and their sera were prepared for chemistry analysis. Data represent mean values and 95% confidence intervals. ALT = Alanine transaminase; AST = Aspartate aminotransferase; BUN = Blood urea nitrogen; Cr = Creatinine.

## Discussion

The above study demonstrated that BENC-511 was significantly improved in terms of its activity to suppress PI3K/AKT activation, to induce MM cell apoptosis, and to delay tumor growth *in vivo*. Because of the minimal toxicity and great potency in the treatment of MM *in vitro* and *in vivo*, BENC-511 has a great potential for MM treatment.

S14161 has been demonstrated as a pan-PI3K inhibitor, which has no effects on PI3K associated enzymes including AKT, mTOR, PDK1 or GSK-3β. Cell-based and mice-based studies showed that S14161 could be a good candidate for leukemia and myeloma treatment. However, the presence of the chiral structural 4-fluorophenyl group at the chromene brings more work in its preparation and safety evaluation. Thalidomide, the then-best drug for morning sick, turned to be a teratogen which results in thousands of malformed babies due to the less knowledge in the chiral structure [29-31]. Now it is clear that in the two enatiomers of thalidomide, the “R” enantiomer is a relatively safe drug with sedative attributes, while the “S” enantiomer has devastating effects such as teratogenicity [32,33]. Therefore, to improve the efficacy and to reduce the potential safety issue, we synthesized a series of analogs of S14161. The enzymatic assay revealed that the phenyl group is not important because when the whole fluorophenyl group is removed, the resultant QDF-510 and BENC-511 remain active in suppressing PI3K. Therefore, this structural optimization demonstrated that the 4-fluorophenyl group on chromene is dispensable for this class of PI3K inhibitors.

Beyond our prediction, removal of the phenyl ring increases but not decreases the suppressive activity of S14161 in PI3K inhibition because 4 μM of BENC-511 completely suppresses AKT activation within 24 hours, however, it was not markedly affected by S14161 in the same time frame. In the short-term treatment, a certain level of AKT phosphorylation remains at 100 μM within 2 hours, which is similar to LY294002, the classic pan-PI3K inhibitor [34,35]. In contrast, BENC-511 almost suppressed AKT phosphorylation at 50 μM within 30 minutes.

Although other residues could be phosphorylated in AKT, its activation mainly depends on two sites, T308 and S473 [36], and T308 activation is mediated by PI3K via the phosphatidylinositol 3-kinase-dependent kinase 1 (PDK1) and it leads to activation of mTOR complex 1 (TORC1) in which mTOR activation occurs at S2448. Our study clearly demonstrated that BENC-511 inhibited AKT activation at T308 which suggests that BENC-511 probably inhibits PI3K activity. Phosphorylation on S473 facilitates fully activation of AKT, but BENC-511 can inhibit AKT at both T308 and S473 sites, therefore, BENC-511 fully inhibits AKT activation.

PI3K/AKT is the center node of a pyramid of cell signaling pathways including mTOR, p70S6K, 4E-BP1, and GSK-3β signals. mTOR is a serine/threonine protein kinase that regulates PI3K/AKT signals and is frequently referred to as the PI3K/AKT/mTOR signaling pathway [37]. Investigations on PI3K/AKT signaling found that PI3K/AKT inhibition leads to suppressed activation of its downstream signals mTOR, p70S6K, and 4E-BP1 [15]. BENC-511 has no inhibitory effects on AKT and mTOR in the cell-free based enzymatic assays but potently suppresses AKT and mTOR phosphorylation in cultured cells, suggesting that BENC-511 inhibits PI3K activation. p70S6K is a kinase that activates the S6 ribosomal protein thus inducing protein synthesis [38]. 4E-BP1 is a repressor of protein translation and its phosphorylation lifts its repression function [39]. Both p70S6K and 4E-BP1 phosphorylation are regulated by the PI3K/AKT signaling [39,40]. In contrast to p70S6K and 4E-BP1, GSK-3β is negatively regulated by the PI3K/AKT signaling. The effects of BENC-511 on these signals are consistent with its effect on PI3K/AKT, which further suggests that BENC-511 inhibits the PI3K signaling pathway.

The PI3K/AKT signaling pathway is important for MM cell proliferation, survival and anti-apoptosis, downregulation of PI3K activity leads to MM cell death and decreased proliferation. In agreement with its potent activity on PI3K, BENC-511 is more effective than S14161 in inducing MM cell apoptosis. Notably, this apoptosis induced by BENC-511 could be partly attenuated by PI3K activation upon treatment with IL-6 or IGF-1, two key factors of PI3K signaling stimulation and key survival factors for MM cells, which further demonstrates that BENC-511 induces MM apoptosis by targeting the PI3K signaling pathway. Impressively, the anti-myeloma activity of BENC-511 is also proven in myeloma xenograft models. More than 75% inhibition on MM tumor growth in two different models by oral administration suggests that BENC-511 is highly efficacious. Importantly, activation of PI3K activity indicators AKT, mTOR, and p70S6K are also significantly decreased by BENC-511 in myeloma tumor tissues excised from experimental mice, which was accompanied by Caspase-3 activation. Collectively, all these findings demonstrated that BENC-511 not only induces apoptosis but also delays tumor growth in MM xenografts, which is associated with its inhibition on PI3K signals.

In summary, we developed BENC-511 as a more potent PI3K inhibitor than its parental compound by structural optimization. Because of its minimal toxicity and high efficacy, BENC-511 could be developed as a potent orally active anti-myeloma agent, but further safety evaluation should be performed.

## Materials and methods

### Cell lines

MM cell lines LP1, OCI-MY5, OPM2, and JJN3 were kindly provided by Dr. Aaron Schimmer from Ontario Cancer Institute, Toronto, Canada. RPMI-8226 and U266 were purchased from American Type Culture Collection (Washington, DC, USA). Human bone marrow stromal cell line HS-5 was generously provided by Prof. Lin Yang, the Cyrus Tang Hematology Center, Soochow University. All cell lines were maintained in Iscove’s modified Dulbecco medium (Hyclone), supplemented with 10% fetal bovine serum, 100 μg/ml penicillin, and 100 U/mL streptomycin (Hyclone Laboratories, Logan, UT).

### Preparation of S14161 and its analogs

8-Ethoxy-2-(4-fluorophenyl)-3-nitro-2H-chromene (S14161) was synthesized using the domino oxa-Michael-Henry reactions of salicylaldehyde with β-nitrostyrene [41]. As shown in Figure 1A, WQD-612 had a replacement of the fluoro substituent with a hydrogen atom at the para position of the 2-phenyl ring in S14161, while DQJ-610 and DJY-611 had an electron-withdrawing cyano group and electron-donating methoxy substituent at the same position, respectively. Since S14161 has one chiral center in its structure and it was used as a racemate in its biological studies, we further simplified its structure by removing the 4-fluorophenyl group at the 2-position of the chromene core, which generated the 8-ethoxy-3-nitro-2*H*-chromene (QDF-510) and 6-bromo derivative BENC-511 [41].

### AKT phosphorylation analysis

Multiple myeloma cell lines were maintained overnight in Iscove’s modified Dulbecco’s medium containing 0.5% fetal bovine serum, and treated with 100 μM of S14161 or BENC-511 for 0.5 to 2 hours before stimulation with 100 ng/mL human recombinant insulin-like growth factor-1(IGF-1, PeproTech, Rocky Hill, NJ) or 50 ng/mL of interleukin-6 (IL-6, Novoprotein, Summit, NJ) for 15 minutes before being lysed in a RIPA buffer containing 1 mM orthovanadate [11]. After clarification, cell lysates were subjected to Western blotting analysis with anti-phospho-AKT (S473) or anti-AKT.

### Cell growth and viability

Myeloma cell lines were plated at a density of 1 × 10^4^ cells per well in 96-well plates (Wuxi Nest Biotechnology Co., Ltd, Wuxi, China). Cells were treated with BENC-511 with the increasing concentrations. Cell viability was evaluated by MTT assay as described previously [42].

### Apoptosis assay

PRMI-8226, LP1, OPM2, OCI-MY5 cells were treated with BENC-511 (0.5, 1 μM) or S14161 (1 μM) for 24 hour using DMSO as a control. Apoptosis was measured by staining cells with Annexin V-Fluorescein Isothiocyanate (annexin V-FITC) and propidium iodide (PI, Sigma) according to the manufacturers’ instruction. Stained cells were analyzed on a flow cytometer (FACSCalibur, Becton Dickinson).

### Immunoblotting

Whole cell lysates were prepared as described previously [11]. After proteins were then transferred to polyvinylidene difluoride membranes, the blots were then probed with antibodies including monoclonal PARP, Caspase-3, p-AKT(S473), p-AKT(T308), AKT, p-mTOR(S2448), Raptor, p-P70S6K, P70S6K, p-4E-BP1(S65), 4E-BP1 (all were purchased from Cell Signaling Technology, Inc.). GAPDH was purchased from Abgent. β-actin, anti–mouse immunoglobulin G (IgG) and anti–rabbit IgG horseradish peroxidase conjugated antibody were purchased from R&D Systems.

### Multiple myeloma xenograft models

Human multiple myeloma cells (OPM2 and RPMI-8226) were injected subcutaneously into the right flanks of nude mice (5–6 weeks old, female, Shanghai Slac Laboratory Animal Co. Ltd., Shanghai) respectively. When tumors were palpable, mice were randomly divided into two groups (n = 10/group). One group was given BENC-511 (50 mg/kg body weight) in PBS containing 10% Tween 80 and 10% DMSO daily for 20 days, another group was received the vehicle only. Tumor volumes (tumor length × width^2^ × 0.5236) were measured over time with a caliper [11]. Mouse body weight was also monitored every other day. To analyze protein signals from the tumor tissues at the end of the experiment, tumors were excised and snap-frozen immediately in liquid nitrogen. Tissue samples were then minced and homogenized to extract whole cell lysates. The clarified supernatants were applied for Western blotting analyses using specific antibodies.

### Blood physiochemical assays

At the end of the experiment, whole blood samples were collected from the eyes and were immediately subject to complete blood analysis including the white blood cell (WBC), red blood cell (RBC), platelets (PLT) and haemoglobin (HGB) measurement on an automated hematology analyzer (Sysmex KX-21N, Japan). All samples were analyzed within 30 minutes after collection. At the same time, blood sera were isolated by centrifugation at 3,000 × *g* for 10 minutes and frozen for further analysis. Liver function was evaluated with serum levels of physiochemical indexes including alanine aminotransferase (ALT), aspartate aminotransferase (AST), blood urea nitrogen (BUN) and creatinine (Cr). All biochemical assays were performed using a clinical automatic chemistry analyzer (Suzhou Municipal Hospital, Suzhou, China).

### Statistical analysis

Data are presented as mean values with 95% confidence intervals (CIs) unless otherwise indicated. For *in vivo* studies, the Mann–Whitney rank sum nonparametric method was used to test for differences between treatment groups in the weight of the tumors. The *t* test was used for comparisons of two groups in the in vitro studies. All statistical tests were two-sided, and a *P* value less than 0.05 was considered statistically significant.

## Competing interests

The authors declare that they have no competing interests.

## Authors’ contributions

Participated in research design: KH, ZL, XM, BC. Conducted experiments: KH, XX, GC, YZ, JZ, XD, ZZ, BC. Performed data analysis: KH, XX, XM, ZL. Wrote or contributed to the writing of the manuscript: KH, XM, ZL, BC. All authors read and approved the final manuscript.

## Supplementary Material

Additional file 1: Figure S1 (A) OPM2 cells were treated with increasing concentration of S14161, BENC-512, DQJ-610, DJY-611, WQD-612, QDF-511. Seventy-two hours after incubation, cell growth and viability were measured by the MTT assay. (B) Myeloma (RPMI-8226, JJN3, LP1, OCI-My5, U266, OPM2) cells were treated with BENC-511 with the indicated concentration for 72 hours, cell growth and viability were measured by the MTT assay. Click here for file
