# Supplementary figures and images for: Identification of a promising PI3K inhibitor for the treatment of multiple myeloma through the structural optimization
Source: J Hematol Oncol. 2014 Jan 15;7:9. doi: 10.1186/1756-8722-7-9 (PMC3924225; doi:10.1186/1756-8722-7-9)

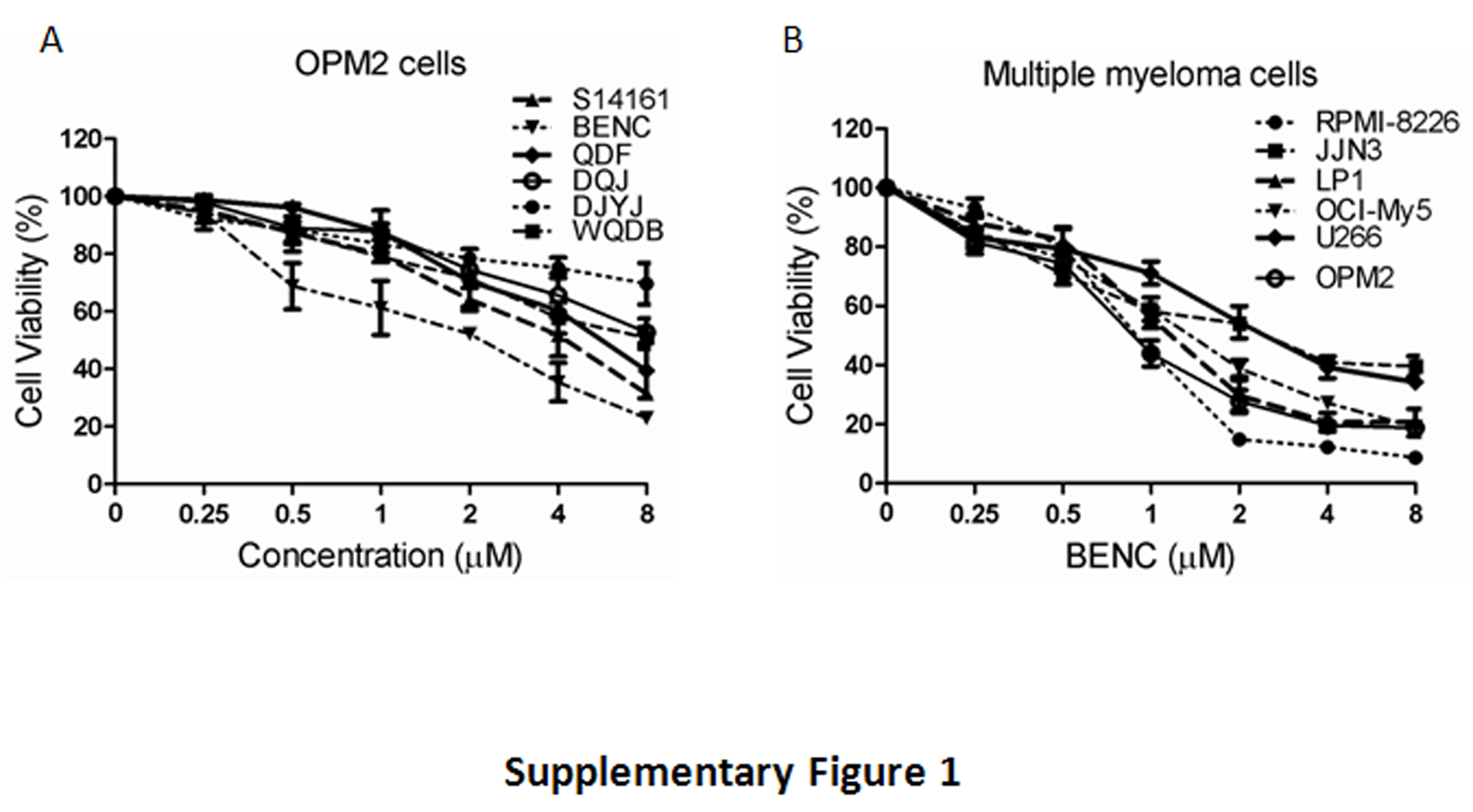

Supplement: Additional file 1: Figure S1 — (A) OPM2 cells were treated with increasing concentration of S14161, BENC-512, DQJ-610, DJY-611, WQD-612, QDF-511. Seventy-two hours after incubation, cell growth and viability were measured by the MTT assay. (B) Myeloma (RPMI-8226, JJN3, LP1, OCI-My5, U266, OPM2) cells were treated with BENC-511 with the indicated concentration for 72 hours, cell growth and viability were measured by the MTT assay. [file 1756-8722-7-9-S1.tiff]
